# The analysis of randomized response “ever” and “last year” questions: A non-saturated Multinomial model

**DOI:** 10.3758/s13428-023-02096-3

**Published:** 2023-05-10

**Authors:** Khadiga H. A. Sayed, Maarten J. L. F. Cruyff, Peter G. M. van der Heijden

**Affiliations:** 1 Department of Methodology and Statistics, Utrecht University, Utrecht, Netherlands; 2 Department of Statistics, Faculty of Economics and Political Science, Cairo University, Cairo, Egypt; 3 Department of Social Statistics and Demography, University of Southampton, Southampton, UK

**Keywords:** Randomized response, Response bias, Efficiency, Goodness-of-fit, Multinomial logistic, Anabolic steroids, Kuk model, Forced response

## Abstract

Randomized response (RR) is a well-known interview technique designed to eliminate evasive response bias that arises from asking sensitive questions. The most frequently asked questions in RR are either whether respondents were “ever” carriers of the sensitive characteristic, or whether they were carriers in a recent period, for instance, “last year”. The present paper proposes a design in which both questions are asked, and derives a multinomial model for the joint analysis of these two questions. Compared to the separate analyses with the binomial model, the model makes a useful distinction between last year and former carriers of the sensitive characteristic, it is more efficient in estimating the prevalence of last year carriers, and it has a degree of freedom that allows for a goodness-of-fit test. Furthermore, it is easily extended to a multinomial logistic regression model to investigate the effects of covariates on the prevalence estimates. These benefits are illustrated in two studies on the use of anabolic androgenic steroids in the Netherlands, one using Kuk and one using both the Kuk and forced response. A salient result of our analyses is that the multinomial model provided ample evidence of response biases in the forced response condition.

**Supplementary Information:**

The online version contains supplementary material available at 10.3758/s13428-023-02096-3.

## Introduction

Participants in sample surveys may face questions about sensitive topics. When such sensitive questions are asked directly, the danger is that respondents either refuse to answer or provide socially desirable answers (Chaudhuri & Mukerjee, 1988). Randomized response (RR) is an interview technique designed to eliminate this evasive response bias (Warner, 1965). This technique utilizes a randomizing device, for instance, a dice or a spinner, to randomly perturb the answers to the sensitive question so that the respondents’ actual status is not revealed. As a result, RR designs protect respondents’ privacy. It has been shown that RR yields more valid estimates than direct questioning, especially when the sensitivity of the behavior of interest increases (Lensvelt-Mulders, Hox, van der Heijden, & Maas, 2005). Since the pioneer work of (Warner, 1965), many extensions and developments have been proposed by various authors. These concern, first, improvements of the Warner design with respect to the statistical efficiency and/or the respondent’s cooperation by modifying the structure, for example, see (Boruch, 1971; Greenberg, Abul-Ela, Simmons, & Horvitz, 1969; Kuk, 1990; Cruyff, Böckenholt, & van der Heijden, 2016; Ulrich, Schröter, Striegel, & Simon, 2012; Gupta, Tuck, Gill, & Crowe, 2013; Lee, Sedory, & Singh, 2013; Su, Sedory, & Singh, 2017; Sayed & Mazloum, 2020; Sedory, Singh, Olanipekun, & Wark, 2020; Reiber, Schnuerch, & Ulrich, 2022; Zapata, Sedory, & Singh, 2022). Second, they concern the improvement of the analysis of RR data by relating the sensitive question measured with RR, with covariates and taking into account the possibility for noncompliance to the instructions of RR design (Scheers & Dayton, 1988; Clark & Desharnais, 1998; Böckenholt & van der Heijden, 2007; Cruyff, van den Hout, van der Heijden, & Böckenholt, 2007; Böckenholt, Barlas, & van der Heijden, 2009; Moshagen, Musch, & Erdfelder, 2012; Hoffmann & Musch, 2016; Reiber, Pope, & Ulrich, 2020; Hoffmann, Meisters, & Musch, 2020; Wolter & Diekmann, 2021; Meisters, Hoffmann, & Musch, 2022b).

RR has been employed to a wide range of sensitive topics, such as illegal drug use, drunk driving, sexuality, tax evasion and the violation of a social norm. The most commonly asked question in RR studies is the “ever” question “Have/did you ever …?”, inquiring about the presence of the sensitive characteristic at some point during the respondents’ life. This question has been used in numerous studies, including those on doping and illicit drug abuse (Striegel, Ulrich, & Simon, 2010; Stubbe, Chorus, Frank, de Hon, & van der Heijden, 2014), rape victimization (Soeken & Damrosch, 1986), induced abortion (Lara, García, Ellertson, Camlin, & Suárez, 2006; Perri, Pelle, & Stranges, 2016; Ghofrani, Asghari, Kashanian, Zeraati, & Fotouhi, 2018), and extradyadic sex (Tu & Hsieh, 2017). Less often interest goes out to the presence of the sensitive characteristic in a recent period. For example, the “last year” question “In the last year, have/did you …?” was asked in studies on doping use (Dietz et al., 2013; Dietz et al., 2018; Ulrich et al., 2018), tax evasion (Korndörfer, Krumpal, & Schmukle, 2014), and disability benefits (Lensvelt-Mulders, van der Heijden, Laudy, & van Gils, 2006).

The present paper introduces a multinomial model for the joint analysis of the “ever” and “last year” questions. The model is based on a compound response variable consisting of the four observed randomized response profiles {*nn*,*ny*,*yn*,*yy*}, with *y* denoting a “Yes” and *n* a “No” response, and the first and second response of each pair referring to the “ever” and “last year” question, respectively. Since the observed responses are randomized, each of these four profiles can occur. The aim of the analysis is to estimate the prevalence of the unobserved true response profiles, i.e. the honest answer that would have been given to direct questions. Based on the true response profiles, we can distinguish the following types of carriers of the sensitive characteristic:

- “never” non-carriers with true response profile *nn*,
- “former” carriers (in the period before last year) with true response profile *yn*,
- “last year” carriers (last year and possibly before) with true response profile *yy*.

Note that the type of carrier with true response profile *ny* does not exist, because it is not possible to have never carried the sensitive characteristic, but to have carried in the last year. Also note that “last year” carriers may or may not have been carriers of the sensitive characteristic in the period before last year, because a true *y* to the “ever” question may refer to both last year and the period before. Obviously, “last year” could be replaced by any recent period, but for ease of reference the phrase “last year” is used.

The joint analysis with the multinomial model has three benefits over two separate analyses with binomial models. Firstly, the multinomial distinguishes a “former” category directly and more precisely than the binomial model. The reason for this is that the compound response variable of the multinomial model takes the within-subject character of the two responses into account, while the separate analyses of the two response variables with the binomial model does not. Additionally, estimating the “former” category as the difference between the two separate estimates of “ever” and “last year” is less efficient than the multinomial estimate for this category. The “former” category is especially useful for assessing the efficacy of an intervention program, like for example an anti-doping policy, because it is an indication of the number of doping users that have stopped using during the last year. Furthermore, the analysis of the compound response variable precludes the illogical result $\hat \pi _{last~year} > \hat \pi _{ever}$ that may occur when analyzing both response variables separately. A second benefit of the multinomial model is an efficiency gain; a study presented later in this paper shows that the multinomial model estimates the prevalence of the “last year” category one-and-a-half to three times more efficiently than the binomial model. Thirdly, since the multinomial estimates three true state probabilities from four observed response profile frequencies, the model has one degree of freedom that can be used to perform a goodness-of-fit test to detect response biases.

Besides the prevalence estimates, it is also important to investigate potential effects of covariates on the prevalence estimates of “never”, “former” and “last year” categories. In line with the extension of other RR models with regression components (Cruyff, Böckenholt, van der Heijden, & Frank, 2016), we derive a multinomial logistic regression model. The choice for the multinomial logistic regression model is motivated by the nominal nature of the true states “never”, “former” and “last year”. To facilitate the interpretation of the coefficients of this model, we also derive the marginal effects of the covariates (Wulff, 2015; Onukwugha, Bergtold, & Jain, 2015). In this paper, we present two studies on doping use by (Duiven & de Hon, 2015) and (Hilkens, Cruyff, Woertman, Benjamins, & Evers, 2021) in which the “ever” question was asked in conjunction with the “last year” question. To the best of our knowledge, these are the only two studies that have done so.

The paper is structured as follows. The next section provides a brief description of two data sets concerning the use of anabolic androgenic steroids in the Netherlands; one among male gym users and the other among elite status athletes. The model section derives the multinomial model and its extension to a logistic regression model, and includes a power study and the derivation of the marginal effect for the latter. The results section presents the analysis of both data sets. The discussion section ends the paper with a summary of the main results and some concluding remarks.

## The data

Data to illustrate the benefits of the multinomial model are from two independent surveys. Both surveys assess the use of anabolic androgenic steroids in the Netherlands. Survey I was conducted by the Anti-Doping Authority Netherlands (Duiven & de Hon, 2015) and Survey II by HAN University of Applied Sciences and Utrecht University (Hilkens et al., 2021).

In survey I, data were collected from 1,053 Dutch elite athletes. The sample of the Utrecht University study consists of 2,272 male gym users, aged between 18-40 years, who perform resistance fitness. In both surveys the respondents are asked the following two questions concerning the use of anabolic steroids:

- Have you ever used anabolic steroids (e.g., Testosterone, Deca, Winstrol, Dianabol, Anavar)?
- Have you used anabolic steroids (e.g., Testosterone, Deca, Winstrol, Dianabol, Anavar) in the last year?

The participants in survey I are instructed to use two digital dice for the “ever” question and another two for the “last year” question. After reading the question, they are asked to press “enter” to stop the dice and to calculate the sum of the dice. By pressing “enter” again, the sensitive question with the answers appears on the screen. The survey had an experimental design, with random assignment to either the Kuk condition (Kuk, 1990) (*n* = 515) or the forced response condition (Boruch, 1971) (*n* = 538).

The forced response condition works as follows:

- If the sum of the dice is 2, 3 or 4, athletes are instructed to answer the question with “Yes”.
- If the sum of the dice is 10, 11 or 12, they are instructed to answer “No” .
- If the sum is 5, 6, 7, 8 or 9, they are instructed to give a truthful answer (“Yes” or “No”).

As the probability of 2, 3 or 4 and of 10, 11 or 12 is 1/6, and the probability of 5 to 9 is 4/6, the probability that the randomized response coincides with the true response is 5/6.

In the Kuk condition the sensitive question is answered with the letters “A” or “B”. The meaning of these letters depends on the outcomes of dice:

- If the sum of the dice ranges from 2 to 9, the letter “A” refers to “Yes” and “B” to “No”.
- If the sum of the dice is 10, 11 or 12, the letter “A” refers to “No” and “B” to “Yes”.

The Kuk answer scheme also results in a probability of 5/6 that the randomized response coincides with the true response.

The advantage of the forced response technique is that, with the majority of the outcomes of the dice (six possibilities out of eleven) resulting in a forced response while the true probability of a forced response is only 1/3, respondents feel safer than they actually are (Fox & Tracy, 1986). A disadvantage of forced response is that a “Yes” response is obviously incriminating, and that respondents may not want to incriminate themselves by giving either a forced or an unforced “Yes” answer (Boeije & Lensvelt-Mulders, 2002).

The key idea of the Kuk technique is to avoid the incriminating responses by using neutral answers like the color of the card or the letters “A” or “B”. By using neutral answer categories, the expectation is that respondents are more willing to follow the RR procedure.

In survey II, the respondents are presented with a screen showing the sensitive question and a circle and a square. After reading the question, they click the “Start” button , and the words “Yes” and “No” alternately appear in either the circle or the square. When they click the “Stop” button, the alternation is stopped, and the respondents are asked to either answer “circle” or “square”, depending on where their true response ended up. The probability that the ”Yes” response ends up in the circle is fixed at 5/6. So as in Study I, the probability that the randomized response coincides with true response is 5/6.

In both surveys, a practice question preceded the sensitive question to ensure that respondents understood the instructions properly. Several precautions were taken to guarantee confidentiality and anonymity. In Survey I, the online questionnaire was made available via an anonymous link, and participants were informed that the questions were framed in such a way that the answers could not be traced back to individuals, and that the data is processed confidentially by Utrecht University and would not be made available to the Anti-Doping Authority Netherlands. In Survey II, participants were informed that the data is processed and analyzed confidentially and anonymously, and that the outcome of the randomizer is unknown to the researchers. Ethical approval for the secondary analysis of the data was obtained from the Ethics Committee of the Faculty of Social and Behavioural Sciences of Utrecht University.

## The models

In this section we derive the multinomial model for prevalence estimation, and the multinomial logistic model for regression analysis. We start with a review of the binomial model for a single dichotomous RR question.

### Binomial model

Let $\pi ^{*}_{j}$ denote the probability of observing a randomized response *j* and let *π*_*s*_ be the probability of a true response *s*, for *j*,*s* ∈{*n*, *y*}. The binomial model for this design is given by:

1 $$ \pi^{*}_{j} = \sum\limits_{s} p_{j|s} \pi_{s}, \ \ \ \ p_{j|s}\neq0.5, $$

where *p*_*j*|*s*_ is the conditional probability of observing the randomized response *j* given the true response *s*, as determined by the randomizer. The model can be presented in a more concise manner in matrix notation by ***π***^∗^ = ***Pπ***, i.e.,

2 $$ \left( \begin{array}{c} \pi^{*}_{n}\\ \pi^{*}_{y} \end{array} \right)= \left( \begin{array}{cc} p_{n|n} & p_{n|y} \\ p_{y|n} & p_{y|y} \end{array} \right) \left( \begin{array}{c} \pi_{n}\\ \pi_{y} \end{array} \right) . $$

The parameter vector ***π*** can be estimated either with the moment estimator $\hat {\boldsymbol \pi }=\boldsymbol {P}^{-1}\boldsymbol {\hat \pi }^{*}$, where $\boldsymbol {\hat \pi }^{*}$ is a vector with the relative randomized response frequencies, or by maximization of the log likelihood function $\ln \ell (\boldsymbol {\pi \mid } \boldsymbol {n})={\sum }_{j} n_{j} \ln \left ({\sum }_{s} p_{j\mid {s}}\pi _{s}\right )$. Both methods yield identical estimates and a perfect fit when the parameter estimates are within the parameter space (0,1). When $\hat \pi _{y}^{*}<p_{y\mid n}$, however, the moment estimator yields $\hat \pi <0$ and a perfect fit, while the maximum likelihood estimator yields the boundary solution $\hat \pi =0$ with a goodness-of-fit statistic $G^{2}_{(0)}>0$, where $G^{2}_{(df)}=2{\sum }_{j}n_{j}\ln n_{j}/\hat {n}_{j}$ with *n*_*j*_ and $\hat {n}_{j}$ respectively denoting the observed and fitted response frequencies. The latter result is unexpected, as generally $G^{2}_{(0)}=0$. Such a result can either be due to chance (*π*_*y*_ close to zero and the randomization resulting in less “Yes” responses than expected on the basis of *p*_*y*|*y*_ and *p*_*y*|*n*_), or to evasive response bias (van den Hout & van der Heijden, 2004).

### Multinomial model

Now consider a multinomial model for two dichotomous RR questions, each asking about a different sensitive characteristic. Let *j* and *k* denote the respective randomized responses to the first and second question, and *s* and *t* the respective true response profiles, for *jk*,*st* ∈{*nn*,*yn*,*ny*,*yy*}. Then

3 $$ \pi^{*}_{jk} = \sum\limits_{st} p_{jk|st} \pi_{st}, $$

where *p*_*jk*|*st*_ = *p*_*j*|*s*_*p*_*k*|*t*_ is the conditional probability of observing a randomized response profile *jk* given a true response profile *st*, assuming that the randomizer is applied independently to both questions (Chaudhuri & Mukerjee, 1988; van den Hout & van der Heijden, 2002). In matrix notation

4 $$ \left( \begin{array}{c} \pi^{*}_{nn}\\ \pi^{*}_{ny}\\ \pi^{*}_{yn}\\ \pi^{*}_{yy} \end{array} \right)= \left( \begin{array}{cccc} p_{nn|nn} & p_{nn|ny} & p_{nn|yn}& p_{nn|yy}\\ p_{ny|nn} & p_{ny|ny} & p_{ny|yn}& p_{ny|yy}\\ p_{yn|nn} & p_{yn|ny} & p_{yn|yn}& p_{yn|yy}\\ p_{yy|nn} & p_{yy|ny} & p_{yy|yn}& p_{yy|yy} \end{array} \right) \left( \begin{array}{c} \pi_{nn}\\ \pi_{ny}\\ \pi_{yn}\\ \pi_{yy} \end{array} \right) . $$

The model of Eq. 3 is not appropriate for the “ever” and “last year” questions, since these questions concern the same sensitive characteristic. To emphasize the difference, we replace the true response profiles *st* of Eq. 3 by the true state categories *r*, for *r* ∈{*nn* ≡“never”,*yn* ≡“former”,*yy* ≡“last year”}. The true response profile *ny* = *∅* (i.e., never having had the sensitive characteristic, but having had it during the last 12 months) is no part of *r*, since it cannot occur in practice. The multinomial model for the “ever” and “last year” questions is then given by:

5 $$ \pi^{*}_{jk} = \sum\limits_{r} p_{jk|r} \pi_{r}, $$

where *p*_*jk*|*r*_ is the conditional probability of observing a randomized response profile *jk* given a true state category *r*. In matrix notation we have

6 $$ \left( \begin{array}{c} \pi^{*}_{nn}\\ \pi^{*}_{ny}\\ \pi^{*}_{yn}\\ \pi^{*}_{yy} \end{array} \right)= \left( \begin{array}{ccc} p_{nn|never} & p_{nn|former} & p_{nn|last~year}\\ p_{ny|never} & p_{ny|former} & p_{ny|last~year}\\ p_{yn|never} & p_{yn|former} & p_{yn|last~year}\\ p_{yy|never} & p_{yy|former} & p_{yy|last~year}\ \end{array} \right) \left( \begin{array}{c} \pi_{never}\\ \pi_{former}\\ \pi_{last~year} \end{array} \right) . $$

Since the model of Eq. 5 estimates three true state categories from four randomized response profiles, it has one degree of freedom. In the next two subsections we show how this degree of freedom can be used to test the goodness-of-fit of the model, and that the model enhances the efficiency of the estimator $\hat \pi _{last~year}$.

### Multinomial logistic regression model

The extension of the multinomial model of Eq. 5 to a regression model requires that the probabilities *π*_*r*_ be expressed as a logistic function of the covariates. Let $\boldsymbol {x}_{i} = (1, x_{i1}, \dots , x_{ip})'$ be a vector with covariates for individual $i = 1,2{\dots } n$, and $\boldsymbol {\beta }_{r}=(\beta _{0r}, \beta _{1r}, \dots ,\beta _{pr})'$ the vector with the regression coefficients for category *r*. With “never” as the reference category, ***β***_*never*_ = ***0***, we have

7 $$ \begin{array}{@{}rcl@{}} \pi_{ir}&=&\frac{\exp(\boldsymbol{\beta}^{\prime}_{r} \boldsymbol{x}_{i})}{{\sum}_{h=1}^{3} \exp(\boldsymbol{\beta}^{\prime}_{h} \boldsymbol{x}_{i})},\\ && r, h\in\{1 = \text{never}, 2=\text{former}, 3 = \text{last year}\}, \end{array} $$

rendering the multinomial logistic regression model

8 $$ \pi^{*}_{ijk} = \sum\limits_{r=1}^{3} p_{jk|r} \pi_{ir}, \qquad jk\in\{nn, ny, yn, yy\}. $$

#### Estimation and goodness-of-fit

The maximum likelihood estimates (MLEs) of regression coefficients ***β***_*r*_ are obtained by maximization of the kernel of the log likelihood function

9 $$ \ln\ell(\boldsymbol\beta\mid \boldsymbol{ x}_{i})=\sum\limits_{i=1}^{n}\sum\limits_{jk}\ln\pi^{*}_{ijk}. $$

After plugging $\hat {\boldsymbol {\beta }_{\boldsymbol {0}}}$ in Eq. 8, the intercept-only version of Eq. 9 yields the MLE $\hat {\boldsymbol {\pi }}$ of ***π*** = (*π*_*never*_,*π*_*former*_,*π*_*lastyear*_)^′^ of Eq. 5. The sampling variances of $\hat {\boldsymbol {\pi }}$ can either be obtained with the delta method, or analytically by the variance equations presented in AppendixA. The goodness-of-fit of this model can be investigated with the *G*^2^ statistic with one degree of freedom.

The likelihood ratio test statistic (LR) can be used to test the significance of a model with covariates. The test statistic is twice the difference between the log likelihoods of the fitted model and the intercept-only model, and has a chi-squared distribution with 2*p* degrees of freedom: the number of covariates *p* multiplied by number of categories of the dependent variable minus 1.

### Marginal effects

Coefficients of the multinomial logistic model are not easy to interpret. One reason is that their interpretation in terms of logodds or odds ratios does not provide insight into the direction nor the magnitude of a covariate’s effect on the probability of a specific outcome (Wulff, 2015). For instance, a negative coefficient in the vector $\hat {\boldsymbol \beta }_{last~year}$ means that for an increase in the value of a continuous covariate, the odds to belong to the “last year” category decreases relative to odds to belong of the baseline category “never”, but this does not necessarily imply that the probability of “last year” category also decreases, nor does it provide insight in the change in the probability in terms of percentage points. Furthermore, the magnitude and statistical significance of the coefficient of the interaction term can not be used to assess the interaction effect for logistic models (Ai & Norton, 2003). Therefore we make use of marginal effects: the estimated effect of a unit change of a covariate on the estimated probabilities of the model outcomes (Wiersema & Bowen, 2009).

The marginal effects of a continuous covariate measure the instantaneous change of the response variable for a unit change in the covariate while holding the other covariates constant. For a multinomial logistic regression model without interaction or higher ordered terms, the marginal effects of a continuous covariate *p* for the outcome *r* are (Greene, 2003)

10 $$ \begin{array}{@{}rcl@{}} \text{ME}_{ipr}&=&\frac{\partial\pi_{ir}}{\partial x_{ip}}= \pi_{ir}(\beta_{pr}-\sum\limits_{h=1}^{3} \pi_{ih} \beta_{ph}),\\ && r, h\in\{1 = \text{never}, 2=\text{former}, 3 = \text{last year}\}.\\ \end{array} $$

This formula shows that, through the probabilities *π*_*ir*_, the marginal effects of covariate *p* depend on the values of all other covariates in the model and its sign may change across the range of the covariate of interest. For a categorical covariate, marginal effects show the difference in the predicted probabilities for one category relative to the reference category.

Average marginal effects (AMEs) are obtained by averaging the individual marginal effects over (a subset of) the observations. AMEs provide insight in the average effect of a covariate on the predicted probabilities of the categories of the dependent variable in the sample or within a subgroup of the sample.

The existing statistical packages, e.g., margins (Leeper, Arnold, Arel-Bundock, & Long, 2021) for calculating marginal effects of the multinomial logistic model do not account for RR perturbation. Our R package RRmultinom for the estimation of the multinomial logistic RR regression model and the marginal effect is available at the github page https://github.com/Khadiga-S/RRmultinom.git.

### Power study

In this section, we compare the efficiency and power of the binomial model of Eq. 1 and the multinomial model of Eq. 5 with respect to the estimators $\hat \pi _{last~year}$ and $\hat \pi _{former}$. For both models, the conditional probabilities *p*_*y*∣*y*_ and *p*_*n*∣*n*_ are set to 5/6, as in the two applications.

**Fig. 1:**
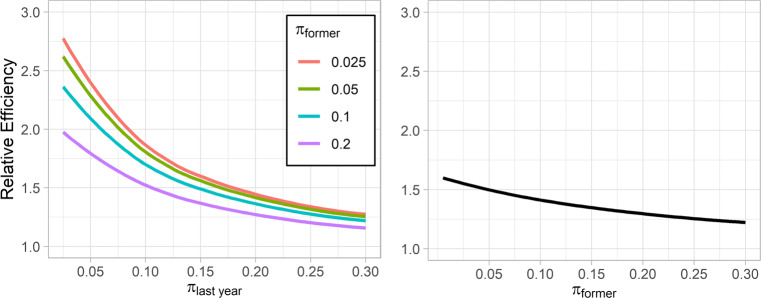
Relative efficiencies of the estimators $\hat \pi _{last~year}$ (left panel) and $\hat \pi _{former}$ (right panel) of the multinomial model with respect to the binomial model, for different values of *π*_*former*_ and *π*_*lastyear*_

The left panel of Fig. 1 shows the relative efficiency (RE) curves for

11 $$ \text{RE}(\hat{\pi}_{last~year}) = \frac{\text{var}_{binom}(\hat{\pi}_{last~year})}{\text{var}_{multinom}(\hat{\pi}_{last~year})} $$

with *π*_*lastyear*_ in the interval (0,0.30), *π*_*former*_ ∈{.025,.05,.1,.2} for the multinomial model, and with $\text {var}_{binom}(\hat {\pi }_{last~year})$ and $\text {var}_{multinom}(\hat {\pi }_{last~year})$ respectively denoting the analytical sampling variances of the bi- and multinomial model (for the derivation of these variances, see Appendix file A). The curves show that the multinomial model is more efficient in estimating the prevalence of *π*_*lastyear*_ for smaller values of *π*_*former*_ (and hence larger values of *π*_*never*_), and that it is two to almost three times more efficient than the binomial model when *π*_*lastyear*_ approaches zero, and about 1.3 to 1.4 times more efficient when *π*_*lastyear*_ approaches 0.3. The right panel depicts the RE curves for

12 $$ \text{RE}(\hat{\pi}_{former}) = \frac{\text{var}_{binom}(\hat{\pi}_{former})}{\text{var}_{multinom}(\hat{\pi}_{former})} $$

with *π*_*former*_ in the interval (0,0.30), *π*_*lastyear*_ ∈{.025,.05,.1,.2}, and with $\text {var}_{binom}(\hat {\pi }_{former})$ and $\text {var}_{multinom}(\hat {\pi }_{former})$ respectively denoting the analytical sampling variances of the bi- and multinomial model (presented in Appendix file A). The curves show that the multinomial model is more efficient than the binomial model for smaller values of *π*_*former*_ irrespective of the values of *π*_*lastyear*_ (i.e., smaller and larger values of *π*_*lastyear*_ have no noticeable effect on the RE curves, which are almost the same for each value of *π*_*lastyear*_). Specifically, it is about 1.6 times more efficient than the binomial model when *π*_*former*_ approaches zero, and about 1.2 times more efficient when *π*_*former*_ approaches 0.3. The RE curves for $\hat {\pi }_{never}$ are not displayed, since both models estimate *π*_*never*_ with the same efficiency.

We now consider the (statistical) power, defined as the probability to reject the null hypothesis *H*_0_ : *π*_*lastyear*_ = *π*_0_ given that the alternative *H*_1_ : *π*_*lastyear*_ = *π*_1_ is true. The power is given by:

13 $$ \text{power} = {\Phi}\left( \frac{\pi_{1} - \pi_{0} + z_{\alpha} \sigma_{0}}{\sigma_{1}}\right) $$

where *σ*_0_ and *σ*_1_ are the respective standard deviations of $\hat {\pi }_{recent}$ under *H*_0_ : *π*_*lastyear*_ = 0 and *H*_1_ : *π*_*lastyear*_ ∈{0.025,0.050,0.075,0.100} and *z*_*α*_ is the 100 − *α*^*th*^ percentile of the standard normal distribution (Ulrich et al., 2012). This equation is based on the assumption that the sampling distribution of $\hat \pi _{last~year}$ is approximately normal for sufficiently large *n*.

**Fig. 2:**
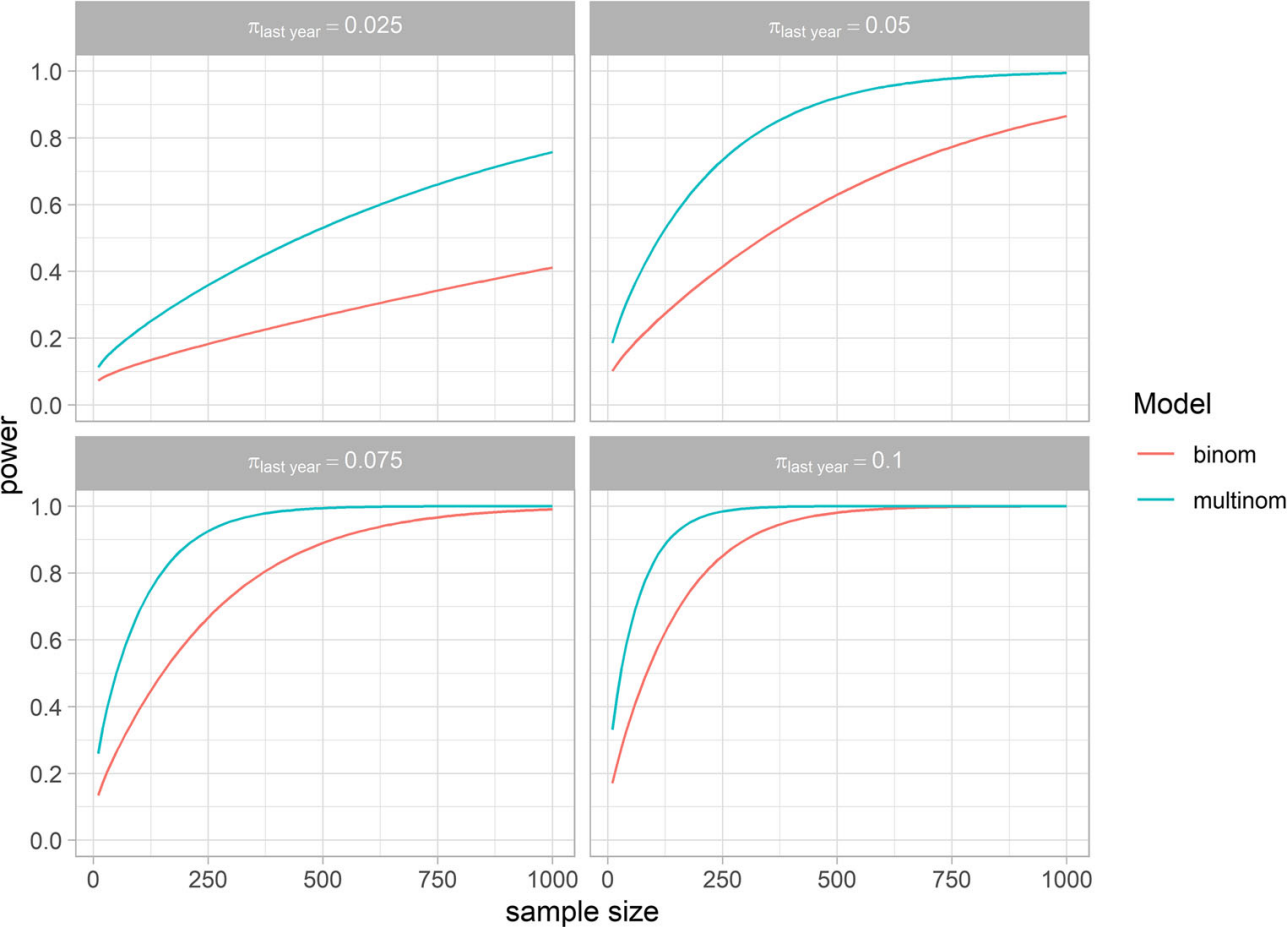
Power curves of the bi- and multinomial model for *π*_*lastyear*_ ∈{0.025,0.05,0.075,0.1} and *π*_*former*_ = 0.1

Figure 2 shows the power curves of both models for the estimator *π*_*lastyear*_ ∈{0.050,0.075,0.100} and *π*_*former*_ = 0.1. The curves show that to attain the desired power of 0.8 for *π*_*lastyear*_ ∈{0.050,0.075,0.100}, the multinomial model requires *n* ≈{270,130,100}, while the binomial model requires *n* ≈{800,350,230}. For *π*_*lastyear*_ = 0.025 and *n* = 1,000, the bi- and multinomial model attains a power of respectively 0.4 and 0.75. As can be derived from Fig. 1, smaller and larger values of *π*_*former*_ would respectively increase and decrease the power of the multinomial model, but these would not affect the power of the binomial model.

## Results

This section presents the prevalence estimates of the use of anabolic steroids of the bi- and multinomial models, and the effects of covariates on these prevalence estimates.

### Prevalence estimates

Table 1 presents the prevalence estimates and 95% confidence intervals for the surveys I and II. For models yielding boundary solutions the confidence intervals are not reported, since maximum likelihood theory does not apply. We analyzed the Kuk and forced response conditions of Survey I separately.

**Table 1:** Prevalence estimates and 95% confidence intervals

	Binomial model		Multinomial model				
Survey	Ever	Last year	Last year	Former	Never = 1 - Ever	$G^{2}_{(1)}$	*p*-value
I: FR	0.0 (-, -)	2.0 (0.0, 6.9)	2.1 (0.0, 3.5)	0.0 (-, -)	98.8 (96.5 1.00)	4.10	.043
I: Kuk	5.9 (0.6, 11.1)	0.9 (0.0, 5.8)	2.4 (0.0, 5.4)	3.4 (0.0, 9.1)	94.2 (88.9, 99.4)	0.55	.457
II: Kuk	8.9 (6.4, 11.5)	3.7 (1.2, 6.1)	4.7 (3.1, 6.3)	4.2 (1.5, 6.9)	91.1 (88.5, 93.7)	1.15	.283

In the forced response condition of Survey I, the binomial model yields a boundary solution for the “ever” question $(G^{2}_{(0)} = 1.57)$ (*p*-values are not defined for a chi-squared distribution on zero degrees of freedom), and a prevalence estimate of 2.0% last year users, which implies that no athletes ever used anabolic steroids, while about 2% used last year. The multinomial model also yields a boundary solution with 2.1% last year users and no former users, and exhibits a significant lack of fit $(G^{2}_{(1)} = 4.10, p = .043)$. These results may be explained by the unwillingness of respondents to give a forced or unforced incriminating “Yes” response.

The Kuk condition of Survey I does not yield any boundary solutions. The binomial model estimates that 5.9% ever used, and 0.9% last year users. The multinomial model estimates a total of 5.8% users, of which 2.4% are last year users, and 3.4% former users, and fits adequately $(G^{2}_{(1)}=0.55, p=.457)$.

In Survey II the binomial model estimates a prevalence of 8.9% ever users and 3.7% last year users. The multinomial also estimates 8.9% ever users, of which 4.7% are last year and 4.2% former users. The model exhibits a satisfactory fit $(G^{2}_{(1)} = 1.15, p =.283)$.

Note that while the models yields different estimates for the last year users, the confidence intervals almost completely overlap, so that these differences are not significant. Also note that the confidence intervals for the “ever” category of the binomial model and the “never” category of the multinomial model have same width (aside from some rounding error), indicating that the multinomial model does not estimate the prevalence of this category more efficiently than the binomial model.

### Regression analyses

For both studies, we fitted multinomial logistic regression models to investigate the effects of covariates on the probabilities of last year and former anabolic steroids use. For Survey I we used the covariate *sex* (45% females and 55% males), and we only analyzed the Kuk data given that the forced response condition yields a boundary solution. For Survey II we used the covariate *age* denoting the standardized ages of the gym users ranging from 18 to 40 (*M* = 24, *SD* = 5.6), and *competitor* indicating whether the gym user participates in bodybuilding competitions (2.3% competitors and 97.7% non-competitors).

Table 2 shows that in Survey I there is no evidence that the sex of athletes affects the true state probabilities of using anabolic steroids among Dutch athletes. In Survey II, we fitted two multinomial logistic RR models: one with and one without the interaction term of the two covariates *age* and *competitor*. We only present the results of the latter model, since the interaction was not significant (*LR* = 0.4,*df* = 2,*p* = .823). The parameter estimates of both covariates are significant. When interpreting the coefficients in terms of odds ratios, we see that the odds to be in the last year category instead of the never category are $\exp (3.26)=26$ times higher for competitors than for non-competitors, and that the odds to be in the former category instead of the never category are $\exp (1.9)=6.7$ times higher for competitors than for non-competitors. Age has a similar effect; the odds to be a last year or former user compared to a never user increase with the respective factors $\exp (0.52)=1.7$ and $\exp (0.82)=2.3$ when age increases with one standard deviation. The interpretations in terms of odd ratios, however, does not provide direct insight in the effects of the covariates on the estimated probabilities of last year, former and never users. For this, we use the marginal effects.

**Table 2:** Parameter estimates and standard errors of the regression models

	Survey I (Kuk)		Survey II		
	Intercept	sex(male)	Intercept	Competitor	Age
Last year	− 4.87 (2.73)	1.70 (2.79)	− 3.31^∗∗∗^ (0.24)	3.26^∗∗∗^ (0.46)	0.52^∗∗∗^ (0.15)
Former	− 3.17^∗∗^ (1.09)	− 0.32 (1.79)	− 3.40^∗∗∗^ (0.46)	1.90^∗^ (0.93)	0.82^∗∗∗^ (0.22)

Standard errors in parentheses

^∗^ *p* < .05, ^∗∗^ *p* < .01, ^∗∗∗^ *p* < .001

### Analysis of marginal effects

Table 3 presents the average marginal effects (AMEs) of the covariates of Surveys I and II. To test the statistical significance of AMEs, the test statistic *z* = *AMEs*/*SE*(*AMEs*) is used, where *SE*(*AMEs*) is the standard error of AMEs. For Survey I, the AMEs of sex suggest that the predicted probability of last year use is on average 3.2 percentage points higher for males than for females, and that the respective probabilities of former and never use by females are on average 1.2 and 2.0 percentage points higher than for males, but none of these effects is significant. For Survey II, the respective predicted probabilities of last year and former use are on average 38.6 and 6.8 percentage points higher for competitors than for non-competitors, while the probability that a non-competitor never used is on average 45.4 percentage points higher than that of a competitor. Each standard deviation increase in age respectively adds on average 1.8 and 3.1 percentage points to the predicted probabilities of last year and former users.

**Table 3:** AMEs and standard errors

	Survey I (Kuk)	Survey II				
	Sex(male)	Competitor	Age	Age_*comp*._	Age_*noncomp*._	Δ_*Age*∗*comp*_
Last year	0.032	0.386^∗∗∗^	0.018^∗∗^	0.082	0.016^∗∗^	0.066
	(0.030)	(0.093)	(0.006)	(0.040)	(0.006)	(0.037)
Former	− 0.012	0.068	0.031^∗∗∗^	0.051	0.030^∗∗∗^	0.021
	(0.058)	(0.079)	(0.008)	(0.035)	(0.008)	(0.035)
Never	− 0.020	− 0.454^∗∗∗^	− 0.049^∗∗∗^	− 0.133^∗∗∗^	− 0.047^∗∗∗^	− 0.086^∗∗∗^
	(0.053)	(0.094)	(0.008)	(0.028)	(0.008)	(0.024)

Standard errors in parentheses

^∗^ *p* < .05, ^∗∗^ *p* < .01, ^∗∗∗^ *p* < .001

We also investigate potential interactions. The columns with *age*_*comp*._ and *age*_*noncomp*._ shows the AMEs of age for competitors and non-competitors, and Δ_*age*∗*comp*_ shows the averaged difference between these two groups. For “never” users, a standard deviation increase in age is on average associated with a decrease of 13.3 percentage points to the predicted probabilities for competitors and a decrease of 4.7 percentage points for non-competitors, and the difference − 13.3 + 4.7 = − 8.6 percentage points (*z* = − 3.58,*p* < .001) indicates that getting older has a significantly larger reduction in the predicted probabilities of “never” users for non-competitors than competitors. In other words, there is an interaction effect between *age* and *competitor* on the predicted probabilities of non-users. For last year and former users, the marginal effects of age are on average positively higher for competitors than for non-competitors, but the average differences between the two groups are not significant.

**Fig. 3:**
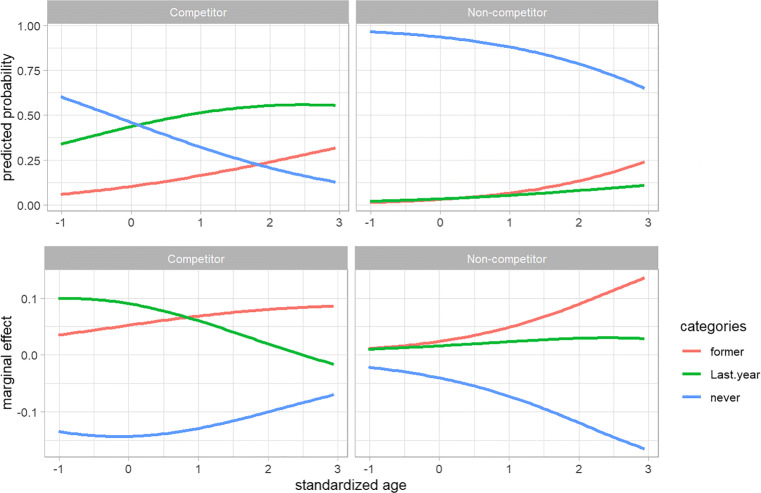
Predicted probabilities and marginal effects of the standardized age of gym users for competitors and non-competitors. Standardized ages on x-axis correspond to ages of 18, 24, 30, 35, and 40 years

Figure 3 shows the predicted probabilities and marginal effects of the three true states as a function of the covariates *competitor* and *age*. The blue lines in the two top panels of the figure show that non-competitors have a much higher probability than competitors to have never used anabolic steroids, and that for both groups this probability decreases with age. The blue lines in the two bottom panels show negative marginal effects for both groups and for all ages, meaning that for both groups the probability to have never used decreases with age. The fact that the slope of the marginal effect is negative for non-competitors and (mainly) positive for competitors indicates that this decrease is stronger for non-competitors than for competitors. For last year use (green lines) the opposite holds. For both groups the predicted probability increases with age, which is reflected by the the positive marginal effects. For competitors, however, the slope of the marginal effect is negative and the marginal effects itself become negative for the standardized ages above 2.5 (corresponding to an age of 35). This means that from that age on, the probabilities of last year use start to decrease. The predicted probabilities of former use (red lines) are slightly smaller for the non-competitors than for competitors and increase with age for both groups, and the marginal effects indicate that the rate of this increase is slightly higher for competitors. The narrow gaps between the two bottom panels for the marginal effects of “former”, and large gaps for “never”, affirm the findings in the last column of Table 3. However, for “last year”, there is a noticeable gap between the two panels for the standardized ages below 2.5 and this gap gets narrower above that age. This motivated us to test how changes from 18 to 35 years old are associated with the predicted probabilities of “last year” for competitors and non-competitors. The results revealed that the AME of an increase in age from 18 to 35 years for competitors is an increase of 8.8 percentage points to the predicted probability of “last year”, whereas the AME for non-competitors is an increase of 1.5 percentage points, and the difference 8.8 − 1.5 = 7.3 percentage points, with a standard error of 3.6 percentage points (*z* = 2.03,*p* = .042) indicates that changes in age from 18 to 35 years has a significantly larger increase in the predicted probabilities of “last year” users for competitors than non-competitors. Such information can not be inferred from an analysis of the coefficients.

## Discussion

This paper introduces a multinomial model for the joint analysis of “ever” and “last year” randomized response questions, and extends it to a multinomial logistic regression model. The analysis of the compound response variable with the multinomial model has three advantages over the separate analyses of the two response variables with the binomial model; (i) it renders a category of former carriers which is not directly available under the binomial model, (ii) it allows to estimate the prevalence of last year carriers more efficiently than the binomial model, and (iii) it has a degree of freedom that allows for a goodness-of-fit test. The extension to a logistic regression enables the inclusion of covariates. We illustrated these benefits for two data sets, one of which including both the forced response and Kuk techniques, and we interpreted the effects of the covariates in the regression analyses in terms of marginal effects.

Because the analysis of the compound response variable by the multinomial model takes the within-subject character of the responses to the “ever” and “last year” questions into account, it is able to estimate the prevalence of “former” users directly, whereas the binomial model infers this estimate indirectly as the difference between the “ever” and “last year” prevalence estimates obtained by two separate analyses. Table 1 shows that, when the prevalence estimates of the binomial model are within the parameter space, the bi- and multinomial models yield practically identical estimates for the “ever” category (for the multinomial model these are the complement of the “ever” estimates), but different estimates for the “former” and “last year” categories. This raises the question whether these latter two categories have different interpretations under the bi- and multinomial models? To answer this question we carried out a simulation study, in which we generated 10,000 pairs of randomized responses to the “ever” and “last year” questions from a sample of size *n* = 1,000, with different combinations of ever users; *π*_*ever*_ ∈{.05,.1,.2,.3,.4} and last year users; *π*_*lastyear*_ ∈{.025,.05,.1,.2} such that *π*_*lastyear*_ < *π*_*ever*_. The design probabilities *p*_*n*|*n*_ and *p*_*y*|*y*_ were set to 5/6. The simulation results show that, on average, both models yield identical, unbiased prevalence estimates of all three true states. Summary statistics of the simulation results can be found in Table B1 of the Appendix file B on OSF (https://osf.io/d8unt/files/osfstorage/63b76e9b202f170a2ba6c994).

A comparison of the Kuk and forced response conditions of Survey I suggests instruction non-adherence in the latter condition, since the binomial model for the “ever” question yields a boundary solution in combination with the goodness-of-fit statistic $G^{2}_{(0)}= 1.57$. This result may either be due to chance or to respondents who evasively answered “No” when “Yes” was required. However, the significance of the goodness-of-fit statistic can not be tested because the binomial model lacks the necessary degrees of freedom. The multinomial model also yields a boundary solution, but now the availability of a degree of freedom allows for a goodness-of-fit test. The significance of this test provides evidence for non-adherence in the forced response condition. Since evasive response behavior results in an inflated percentage of *nn* response profiles and a deflated percentage of *yy* response profiles, we can check whether the misfit is due to evasive responses by comparing the percentages of the *nn* and *yy* response profiles in both conditions. These percentages, which can be found in Table B2 of the Appendix file B on OSF (https://osf.io/d8unt/files/osfstorage/63b76e9b202f170a2ba6c994), are respectively 71.0% and 3.7% in the forced response condition and 67.0% and 4.9% in the Kuk condition, suggesting that the forced response design is more prone to evasive response behavior than the Kuk design. A potential explanation for the (greater) susceptibility of forced response to evasive responses is that non-carriers of the sensitive attribute may refuse to falsely incriminate themselves by giving a forced “Yes” answer (van der Heijden, van Gils, Bouts, & Hox, 2000; Boeije & Lensvelt-Mulders, 2002).

In the literature there are many examples of RR models that, in one way or another, correct the prevalence estimates for response biases, see, e.g., (Clark & Desharnais, 1998; Böckenholt & van der Heijden, 2007; Böckenholt et al., 2009; van den Hout, Böckenholt, & van der Heijden, 2010; Cruyff et al., 2007; Reiber et al., 2020; Meisters, Hoffmann, & Musch, 2022a; Moshagen et al., 2012). Due to its degree of freedom, the multinomial model also allows for such a correction. It would, for example, be possible to include an additional parameter in the model that accounts for self-protective no-sayers, i.e., respondents who answer “No” to each question, irrespective of their true state and of the outcome of the randomizer (Böckenholt & van der Heijden, 2007). Under the multinomial model, self-protective no-saying would result in an overestimation of the “Never” category. However, the self-protective no-sayers assumption originally applies to designs with multiple questions about different sensitive attributes.

As a reviewer rightfully pointed out, there is a risk that asking two questions about the same sensitive attribute decreases the perceived privacy protection, and consequently induces additional self-protective response biases. Whether this is indeed the case remains a topic for future research; a qualitative study like that of Boeije and Lensvelt-Mulders (2002) would be helpful in this respect. In the meantime, extra care should be taken to safeguard respondents’ trust in the method, for example by guaranteeing data anonymization, providing a clear explanation of how RR protects the privacy, and using a validated RR design that does not ask the respondent for false self-incrimination.

A relevant question is whether the *G*^2^ test of the multinomial model is also able to detect other kinds of response biases. One type of response bias that has recently received a lot of attention is random answering (Höglinger & Diekmann, 2017; Walzenbach & Hinz, 2019; Atsusaka & Stevenson, 2021), which may be due to disinterest, inattentiveness or insufficient comprehension of the instructions on the part of the respondents. To check whether the multinomial model is able to detect random answering, we simulated the most extreme scenario in which all respondents answer randomly. In this scenario the expected response profile probabilities are given by the vector ***π***^∗^ = (0.25,0.25,0.25,0.25)^′^. Given these probabilities, the multinomial model yields the prevalence estimates $\hat {\boldsymbol \pi }=(0.409, 0.181, 0.409)'$, which in turn yields the vector with fitted response profile probabilities $\hat {\boldsymbol {\pi }}^{*}=(0.32, 0.12, 0.24,0.32)'$ and a goodness-of-fit test statistic $G^{2}_{(1)}=0.14 n$, where *n* is the sample size. This statistic exceeds the critical chi-squared value of 3.86 for *n* > 26. This example shows that the multinomial model is able to detect random answering in the most extreme scenario. It is difficult to predict how random answering would affect the prevalence estimates in less extreme scenarios, but we conjecture that they would be biased toward the above mentioned prevalence estimates $\hat {\boldsymbol \pi }$ obtained under the most extreme scenario.

The marginal effects have been helpful in interpreting the effects of the covariates. Especially for the effect of the covariate *competitor*, the AMEs have been insightful. Although the odds ratios of 26 and 6.7 for *competitor* indicate a large effect, they leave us in the dark about the effects on the probability scale. The AMEs show that for a competitor the probability to have never used is 45.5 percentage points lower and to have used last year is 38.6 percentage points higher than for non-competitors. Similarly, the plots in Fig. 3 show marginal effects that cannot be inferred from coefficients of the model. For example, while the interaction term of *competitor* and *age* was not significant and therefore not in the model, these covariates interact with respect to the marginal effects; while for both competitors and non-competitors the probability of belonging to the “never” category decreases with age, it does so at a decreasing rate for competitors and an increasing rate for non-competitors. Such additional information helps us to better understand the relationships between variables in the data.

Although this paper discusses “ever” and “last year” questions, the multinomial model is not restricted to this type of questions. Instead of the period in which the sensitive behavior took place, the frequency or severity of the sensitive behavior may be of interest. For example, in case of drunk driving, interest may be in both the occurrence of the behavior as well as in the frequency with which it has occurred. In that case the questions could be “Have you ever driven drunk?” and “Have you driven drunk more than X times?”. Analogously to the “ever” and “last year” questions, the true state profile *ny* cannot occur here. Similarly, in case of the severity of fraud the questions could be “Have you ever committed fraud?” and “Have you earned more than X euros by committing fraud?”. The model, however, seems less suitable for questions about sensitive attitudes or opinions, like for example “Do you think that women possess fewer leadership qualities than men?” (Hoffmann & Musch, 2019).

The multinomial model is also not restricted to the RR designs used in the applications we presented. Since all RR designs with dichotomous questions can be written as the uni- and bivariate models in Eqs. 2 and 4, they can also also be written as the multinomial model of Eq. 6. This includes recent developments like the crosswise and triangular models (Yu, Tian, & Tang, 2008; Hoffmann, Meisters, & Musch, 2021; Sagoe et al., 2021; Meisters, Hoffmann, & Musch, 2020a; Hoffmann & Musch, 2016; Hoffmann et al., 2020), and even the extended crosswise model (Heck, Hoffmann, & Moshagen, 2018; Meisters et al., 2022b; Meisters, Hoffmann, & Musch, 2020b; Mieth, Mayer, Hoffmann, Buchner, & Bell, 2021; Sayed, Cruyff, van der Heijden, & Petróczi, 2022). The latter model consists of two sub-samples with complementary randomization probabilities, and a multinomial model could be formulated for each sub-sample separately, yielding a model with eight observed response profiles and three true response probabilities. Finally, for a design with a dichotomous question about the presence/absence of a sensitive attribute and an ordinal question about the magnitude or severity of that sensitive attribute, an ordinal RR model like the multidimensional model (Cruyff et al., 2016) could be formulated.

## Open Practices Statement

All derivations necessary to reproduce the parameter estimates presented in this manuscript are provided in Appendix file A. Additionally, the R codes necessary to reproduce the results are available online at the github page https://github.com/Khadiga-S/RRmultinom.git

### Electronic supplementary material

Below is the link to the electronic supplementary material.

(PDF 234 KB)

## Data Availability

The data sets analysed during the current study are available on OSF repository (https://osf.io/d8unt/?view_only=3182dfa368414295a6620c0159e180a5).
