# Supplementary material for: The analysis of randomized response “ever” and “last year” questions: A non-saturated Multinomial model
Source: Behav Res Methods. 2023 May 10;56(3):1335–48. doi: 10.3758/s13428-023-02096-3 (PMC10991035; doi:10.3758/s13428-023-02096-3)
Supplement: Supplementary file 1 — (PDF 234 KB) [file 13428_2023_2096_MOESM1_ESM.pdf]

Appendix A: Derivation of sampling variances of $\hat{\pi}_{last\ year}$ and $\hat{\pi}_{former}$ under the binomial and multinomial models

For the binomial model, the sampling variance $\text{var}_{binom}(\hat{\pi}_{last\ year})$ is given by:

$$\text{var}_{binom}(\hat{\pi}_{last\ year}) = \frac{\pi_y^* (1 - \pi_y)^*}{n(p_{y|y} - (1 - p_{n|n}))^2} \tag{1}$$

where $\pi_y^* = p_{y|y}\pi_{last\ year} + (1 - p_{n|n})(1 - \pi_{last\ year})$; is the probability of observing "Yes" answer, $p_{y|y}$ is the transition probability that one who has the sensitive characteristic answers "Yes", and $p_{n|n}$ is the probability of answering "No" for a respondent without the sensitive characteristic. When the transition probabilities $p_{y|y}$ and $p_{n|n}$ are equal, the variance of (1) reduces to the variance under Warner (1965) model and the forced response model. The sampling variance of $\hat{\pi}_{former}$ is calculated as $\text{var}_{binom}(\hat{\pi}_{former}) = \text{var}(\pi_{ever} - \pi_{last\ year}) = \text{var}(\hat{\pi}_{ever}) + \text{var}_{binom}(\hat{\pi}_{last\ year}) - 2cov(\hat{\pi}_{ever}, \hat{\pi}_{last\ year})$, where $cov(\hat{\pi}_{ever}, \hat{\pi}_{last\ year})$ is given by $\pi_{never}\pi_{last\ year}/n$.

For the multinomial design, the variance-covariance matrix of $\hat{\boldsymbol{\pi}} = (\hat{\pi}_{never},\ \hat{\pi}_{former},\ \hat{\pi}_{lastyear})'$, can be obtained from the inverse of the Fisher information matrix:

$$\boldsymbol{I} = -E\left(\frac{\partial^2 \log \ell(\boldsymbol{\pi} \mid \boldsymbol{n})}{\partial \pi_i \pi_j}\right) \tag{2}$$

The kernel of the log likelihood function $\ln \ell(\boldsymbol{\pi} \mid n_{jk}) = \sum_{jk} n_{jk} \ln \pi_{jk}^*$ can be written explicitly

as:

$$\sum_{jk} n_{jk} \ln \pi^*_{jk} = n_{nn} \log \left[(p_{nn|former} - p_{nn|never})\pi_{former} + (p_{nn|last\ year} - p_{nn|never})\pi_{last\ year} + p_{nn|never} \right]$$
$$+ n_{ny} \log \left[(p_{ny|former} - p_{ny|never})\pi_{former} + (p_{ny|last\ year} - p_{ny|never})\pi_{last\ year} + p_{ny|never} \right]$$
$$+ n_{yn} \log \left[(p_{yn|former} - p_{yn|never})\pi_{former} + (p_{yn|last\ year} - p_{yn|never})\pi_{last\ year} + p_{yn|never} \right]$$
$$+ n_{yy} \log \left[(p_{yy|former} - p_{yy|never})\pi_{former} + (p_{yy|last\ year} - p_{yy|never})\pi_{last\ year} + p_{yy|never} \right] \quad (3)$$

By taking the partial derivatives with respect to π_{former} and $\pi_{last\ year}$, we get:

$$\frac{\partial^2 \sum_{jk} n_{jk} \log \pi^*_{jk}}{\partial \pi^2_{former}} = -[n_{nn}(p_{nn|former} - p_{nn|never})^2/{\pi^*_{nn}}^2 + n_{ny}(p_{ny|former} - p_{ny|never})^2/{\pi^*_{ny}}^2$$
$$+ n_{yn}(p_{yn|former} - p_{yn|never})^2/{\pi^*_{yn}}^2 + n_{yy}(p_{yy|former} - p_{nn|never})^2/{\pi^*_{yy}}^2] \quad (4)$$

$$\frac{\partial^2 \sum_{jk} n_{jk} \log \pi^*_{jk}}{\partial \pi^2_{last\ year}} = -[n_{nn}(p_{nn|lastyear} - p_{nn|never})^2/{\pi^*_{nn}}^2 + n_{ny}(p_{ny|lastyear} - p_{ny|never})^2/{\pi^*_{ny}}^2$$
$$+ n_{yn}(p_{yn|recent} - p_{yn|never})^2/{\pi^*_{yn}}^2 + n_{yy}(p_{yy|lastyear} - p_{nn|never})^2/{\pi^*_{yy}}^2] \quad (5)$$

$$\frac{\partial^2 \sum_{jk} n_{jk} \log \pi^*_{jk}}{\partial \pi_{last\ year} \partial \pi_{former}} = -[n_{nn}(p_{nn|last\ year} - p_{nn|never})(p_{nn|former} - p_{nn|never})/{\pi^*_{nn}}^2$$
$$+ n_{ny}(p_{ny|last\ year} - p_{ny|never})(p_{ny|former} - p_{ny|never})/{\pi^*_{ny}}^2 \quad (6)$$
$$+ n_{yn}(p_{yn|last\ year} - p_{yn|never})(p_{yn|former} - p_{yn|never})/{\pi^*_{yn}}^2$$
$$+ n_{yy}(p_{yy|last\ year} - p_{nn|never})(p_{yy|former} - p_{nn|never})/{\pi^*_{yy}}^2]$$

Taking the expectation $E(n_{jk}) = n\pi^*_{jk}$; $jk \in \{nn, ny, yn, yy\}$ and changing the sign, yields the Fisher information:

$$\boldsymbol{I} = n \begin{pmatrix} \sum_{jk}(p_{jk|former} - p_{jk|never})^2/\pi^*_{jk} & \sum_{jk}(p_{jk|former} - p_{jk|never})(p_{jk|last\ \ year} - p_{jk|never})/\pi^*_{jk} \\ \sum_{jk}(p_{jk|former} - p_{jk|never})(p_{jk|last\ \ year} - p_{jk|never})/\pi^*_{jk} & \sum_{jk}(p_{jk|last\ \ year} - p_{jk|never})^2/\pi^*_{jk} \end{pmatrix} \quad (7)$$

Inversion of this matrix yields the variance-covariance matrix:

$$\frac{1}{ndet(I)} \begin{pmatrix} \sum_{jk}(p_{jk|last\ \ year} - p_{jk|never})^2/\pi^*_{jk} & -\sum_{jk}(p_{jk|former} - p_{jk|never})(p_{jk|last\ \ year} - p_{jk|never})/\pi^*_{jk} \\ -\sum_{jk}(p_{jk|former} - p_{jk|never})(p_{jk|last\ \ year} - p_{jk|never})/\pi^*_{jk} & \sum_{jk}(p_{jk|former} - p_{jk|never})^2/\pi^*_{jk} \end{pmatrix} \quad (8)$$

where $det(I) = [\sum_{jk}(p_{jk|former} - p_{jk|never})^2/\pi^*_{jk}][\sum_{jk}(p_{jk|last\ year} - p_{jk|never})^2/\pi^*_{jk}] - [\sum_{jk}(p_{jk|former} - p_{jk|never})(p_{jk|last\ year} - p_{jk|never})/\pi^*_{jk}]^2$. The respective sampling variances of $\hat{\pi}_{former}$ and $\hat{\pi}_{last\ year}$ are the elements on the main diagonal of (8), and the sampling variance of $\hat{\pi}_{never}$ given by the sum of the all elements.
